# Supplementary material for: Real-time monitoring of single ZTP riboswitches reveals a complex and kinetically controlled decision landscape
Source: Nat Commun. 2020 Sep 10;11:4531. doi: 10.1038/s41467-020-18283-1 (PMC7484762; doi:10.1038/s41467-020-18283-1)
Supplement: Supplementary file 1 — Supplementary Information [file 41467_2020_18283_MOESM1_ESM.pdf]

SUPPLEMENTARY INFORMATION

Real-time monitoring of single ZTP riboswitches reveals a complex and kinetically controlled decision landscape

B. Hua, C. Jones, et al.

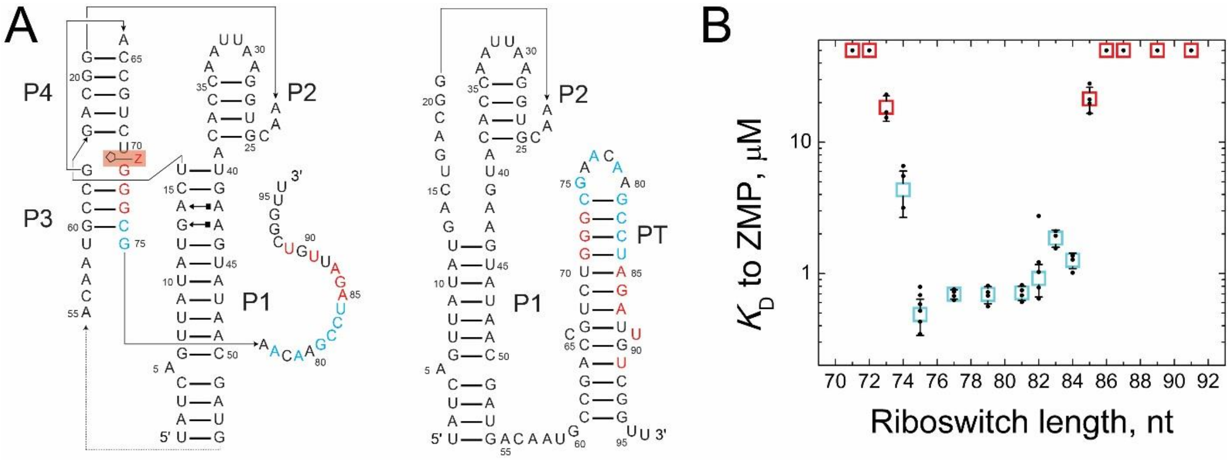

Supplementary Figure 1 ZMP binding to the ZTP riboswitch measured by isothermal titration calorimetry. (**a**) Secondary structures of ZTP riboswitch are shown with the terminator unfolded in the ZMP-bound state (left) and with the terminator (PT) folded in the ZMP-free state (right). Nucleotides are colored to indicate the apparent dissociation constants of the riboswitch variants ending at the respective nucleotides. For example, the 79-nt RNA ending at A79 is colored cyan as this RNA binds ZMP with a K_d of ~1 μM. (**b**) ZTP riboswitch variants transcribed with different 3´-end lengths were thermally folded and titrated with ZMP to determine apparent dissociation constants via isothermal titration calorimetry. RNA lengths for which the apparent dissociation constant was tighter than 5 μM are in cyan, and RNA lengths for which the apparent dissociation constant was weaker than 5 μM or for which binding was not observed (i.e., K_d > 50 μM) are in red. The apparent dissociation constant for the 75-nt RNA is the previously determined value (Jones and Ferré-D'Amaré, NSMB (2015)). Values are mean ± standard deviation (s.d.) shown as large open squares with $n \geq 3$ independent titrations shown as closed circles.

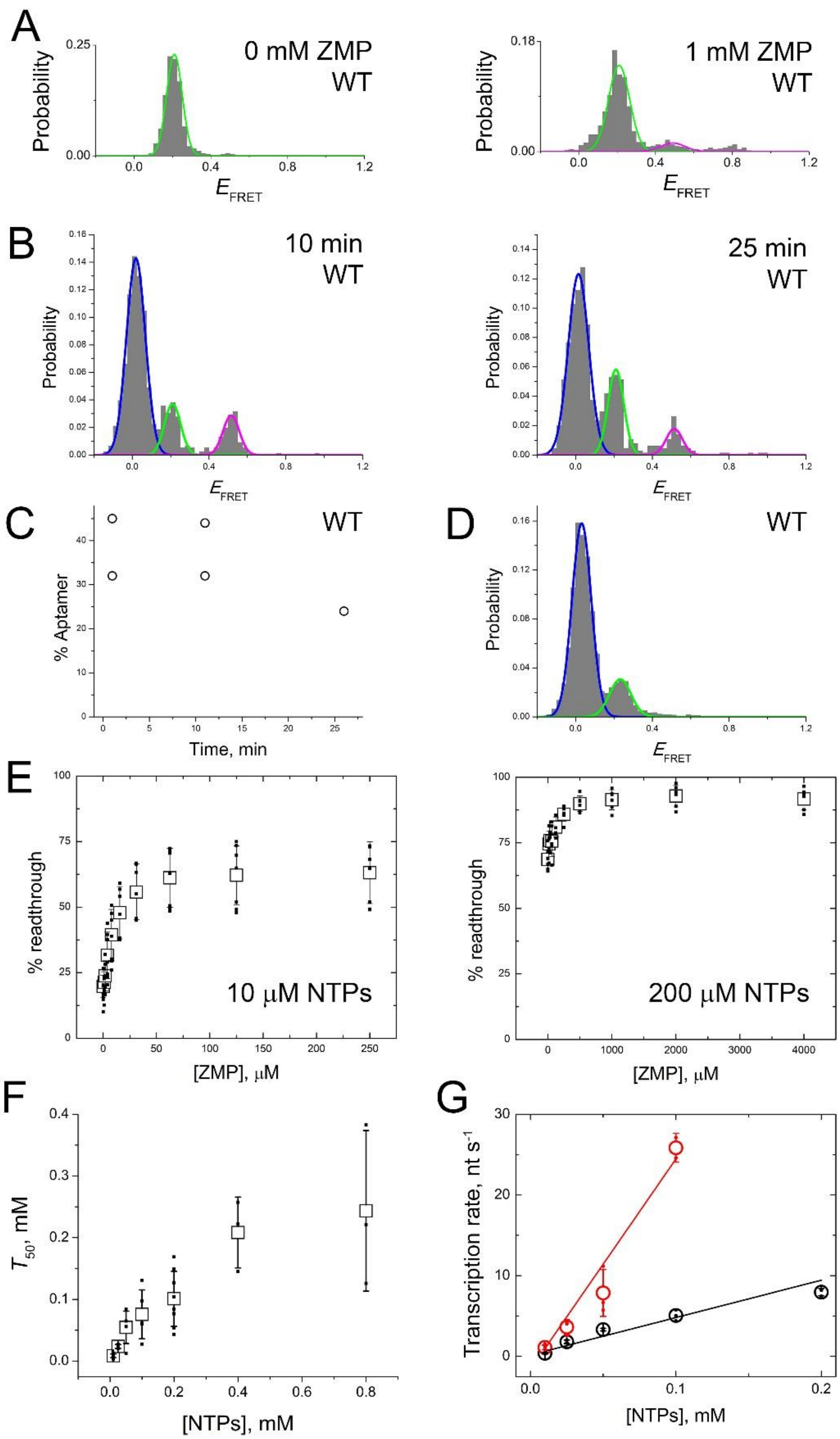

Supplementary Figure 2 Single-molecule assays and bulk transcription experiments of WT ZTP riboswitch. (**a**) The E_{FRET} histograms of WT ZTP riboswitch thermally refolded at 0 mM and 1

mM ZMP. To distinguish the terminator from the ZMP-unbound aptamer, WT was imaged at 1 mM ZMP after being refolded at different ZMP concentrations. (**b**) The E_{FRET} histograms of WT that was vectorially folded at 1 mM ZMP and incubated for 10 min and 25 min before being imaged. (**c**) The remaining percentage of aptamer vs. the post-ATP addition time in VF assays. Each circle represents an individual experiment at the specified time point. The percentage of aptamer is calculated as the ratio of the aptamer population (magenta) over the sum of the terminator (green) and aptamer populations. (**d**) The E_{FRET} histogram of WT that was vectorially folded for 30 s at 0 mM ZMP before 1 mM ZMP was added to stabilize the aptamer conformation. The 30 s was shortened from a longer 1-min folding time that was used in other VF experiments. (**e**) Bulk single-round transcription termination experiments at 0.01 mM NTPs (left) and 0.2 mM NTPs (right) are shown. The transcription midpoints (T_{50}) are determined from fits to these titrations. (**f**) The T_{50} values from bulk single-round transcription termination experiments are shown at different NTP concentrations. (**g**) The apparent rates of RNA synthesis for the terminated (red) and readthrough (black) transcription products are shown at different NTP concentrations. All values in panels **e**, **f**, and **g** are mean ± s.d. shown as large open squares with $n \geq 3$ independent experiments shown as closed squares.

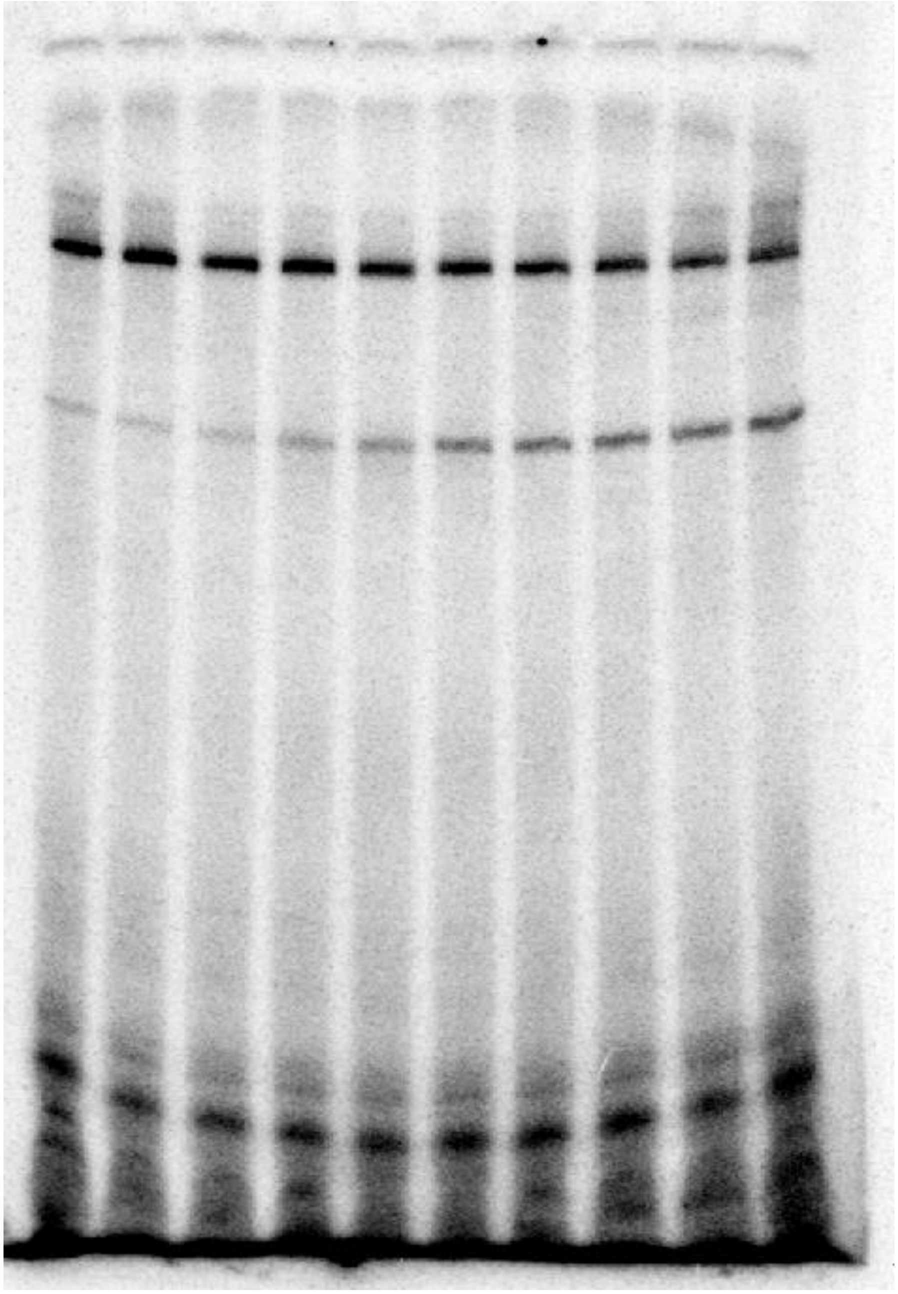

Supplementary Figure 3 Raw gel image of Fig. 3b.

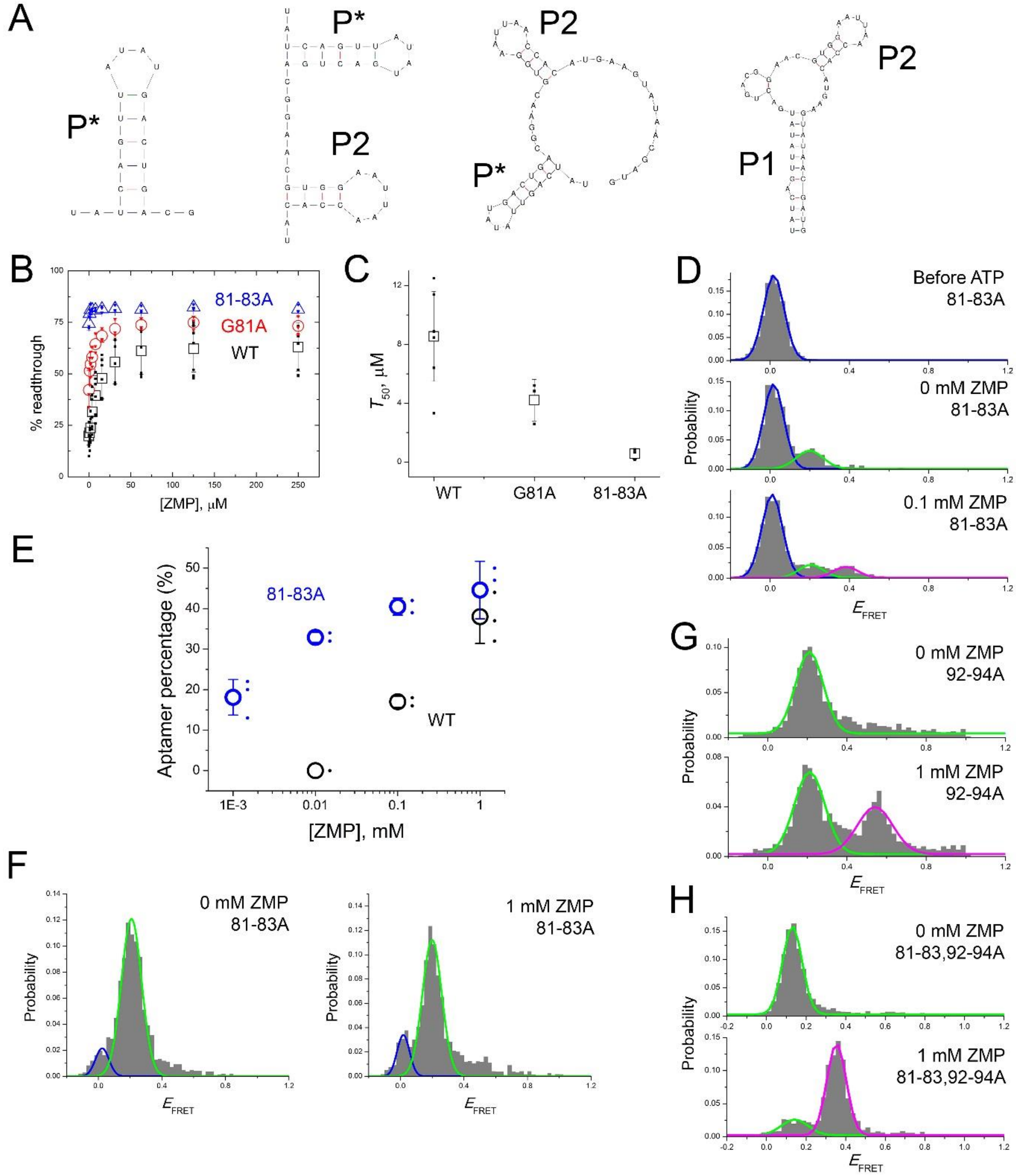

Supplementary Figure 4 Single-molecule assays and bulk transcription experiments of mutant ZTP riboswitches. (**a**) Sequential in silico folding of the ZTP riboswitch. From left to right, the predicted secondary structure of nt 1-20, 1-40, 1-53, and 1-53 (alternative fold) of the WT ZTP

riboswitch sequence. The helix formed by nt 3-8 and 13-18 is labeled P* and is not found in the aptamer fold (Fig. 1). The alternative fold for nt 1-53 contains helices P1 and P2, which are found in the aptamer fold. (**b**) ZTP riboswitch variants containing terminator mutations are titrated with ZMP in single round transcription experiments in the presence of 10 μM NTPs. (**c**) T_{50} values are shown for the terminator variants in **b**. Values for panels **b** and **c** are mean ± s.d. shown as large open symbols with $n \geq 3$ independent experiments shown as closed symbols. (**d**) The E_{FRET} histograms of the 81-83A heteroduplex before and after ATP addition at different ZMP concentrations, as measured by VF with Rep-X. (**e**) The percentage of aptamer fold obtained by VF by Rep-X for WT (black) and terminator mutant 81-83A (blue) in the presence of ZMP (mean ± s.d. shown as large open circles, *n* = 2-3 shown as closed circles and offset for clarity). (**f**) The E_{FRET} histograms of 81–83A thermally refolded at 0 mM (left) and 1 mM ZMP (right). To distinguish the terminator from the ZMP-unbound aptamer, 81–83A was imaged at 1 mM ZMP after being refolded at different ZMP concentrations. Gaussian fitting with global constraints was used to determine the relative population of ssDNA-bound RNA (blue, see also Fig. 2a) and refolded (green). (**g**) E_{FRET} histograms of terminator mutant 92-94A, thermally refolded in the absence of ZMP and imaged at 0 mM (top) and 1 mM ZMP (bottom). Gaussian fitting with global constraints was used to determine the relative population of free (green) and ZMP-bound (magenta) RNA. (**h**) E_{FRET} histograms of terminator mutant 81-83,92-94A, thermally refolded in the absence of ZMP and imaged at 0 mM (top) and 1 mM ZMP (bottom). Gaussian fitting with global constraints was used to determine the relative population of free (green) and ZMP-bound (magenta) RNA.

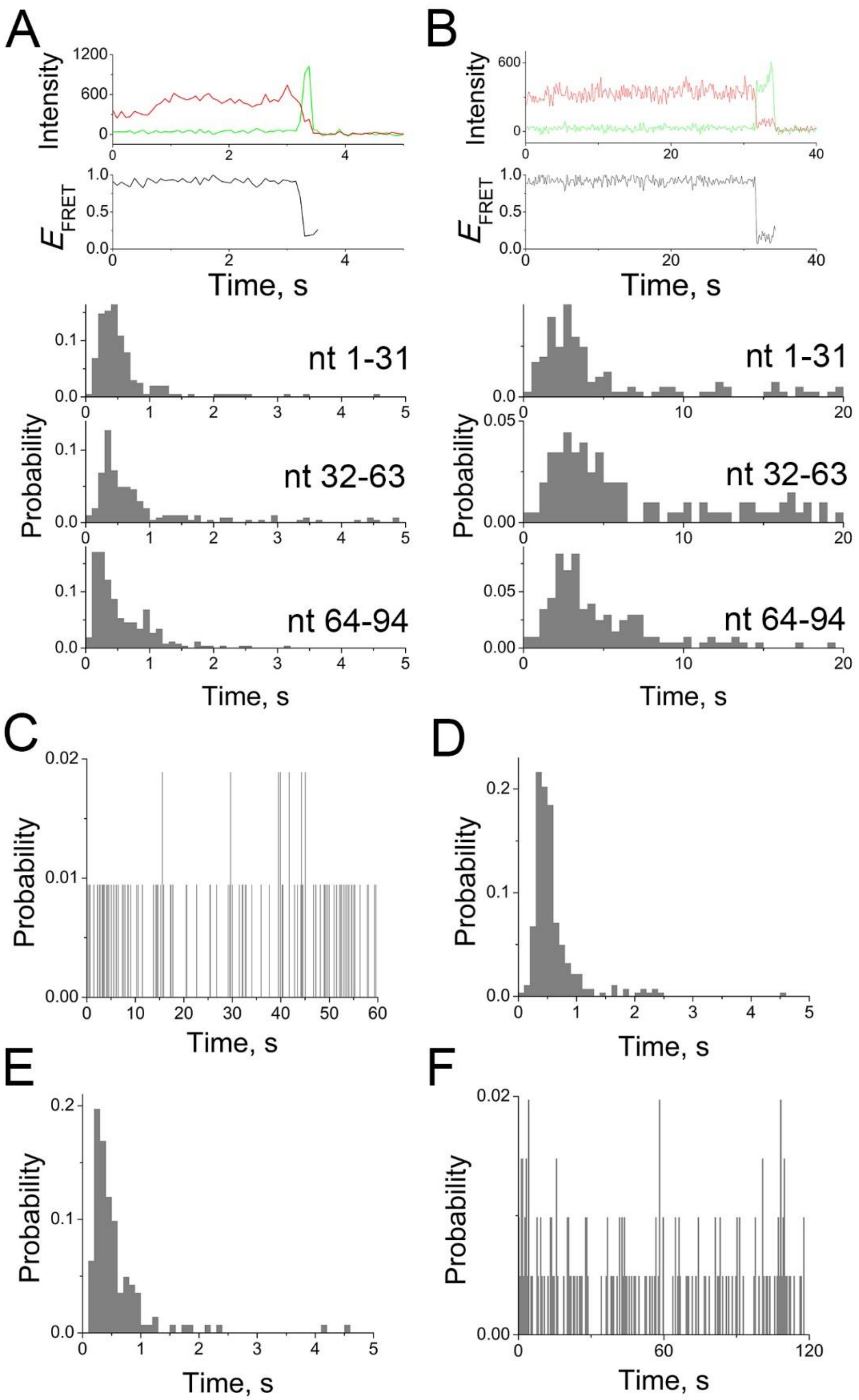

Supplementary Figure 5 Helicase unwinding rate via dropoff measurements. (**a**) Individual single-molecule trajectory (top) and histogram of all dropoff unwinding events (bottom) for Rep-

X unwinding of indicated segments of the ZTP riboswitch (see Online Methods for Δt calculation). (**b**) Individual single-molecule trajectory (top) and histogram of all dropoff unwinding events (bottom) for PcrA-X unwinding of indicated segments of the ZTP riboswitch. Intensity dropoff experiments to measure helicase unwinding rate. See Figure 4a (right) for overall experimental design, in which unwinding causes a loss in FRET followed by a loss in Cy3 fluorescence as labeled oligos dissociate after unwinding. (**c**) Observation of photobleaching of hybrid duplex in the absence of ATP for Rep-X. (**d**) Histograms of time differences (Δt) for all molecules for a Cy5-labeled DNA oligo and Cy3-labeled RNA oligo. (**e**) Histograms of Δt for all molecules for a Cy5-labeled RNA oligo and Cy3-labeled DNA oligo. (**f**) Observation of photobleaching of hybrid duplex in the absence of ATP for PcrA-X.

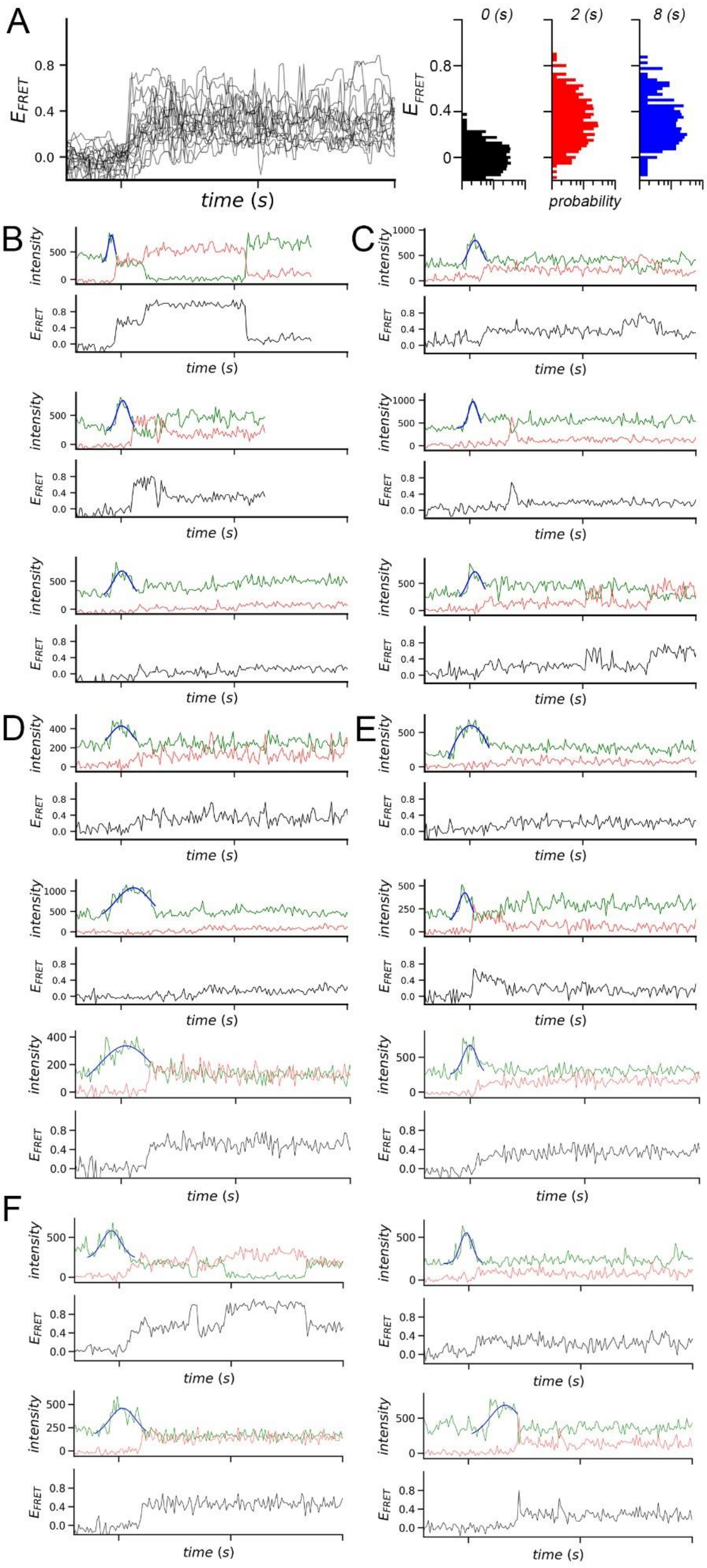
A
B
C
D
E
F
E_{FRET}
time (s)
0 (s)
2 (s)
8 (s)
probability
intensity

Supplementary Figure 6 Real-time observation of heteroduplex unwinding and riboswitch folding in VF assays. (**a**) Real-time trajectories of terminator mutant 81–83A in the presence of 1 mM ZMP using VF with Rep-X, showing all trajectories overlaid (left, *n* = 19), and histograms of all trajectories at 0, 2, and 8 s after PIFE (right). Trajectories are synchronized according to the PIFE peak center at position U32. The time duration between ticks is 5 s. Histogram probabilities are on a log scale, with ticks of 0.1, 1, and 10 percent. (**b**) Example real-time trajectories of WT, and (**c**) terminator mutant 81–83A using VF assays with Rep-X in the absence ZMP. (**d**) Example real-time trajectories of WT and (**e**) 81-83A using VF with Rep-X in the presence of 1 mM ZMP. (**f**) Example real-time trajectories of WT using VF with Rep-X in the presence of 0.01 mM ZMP.

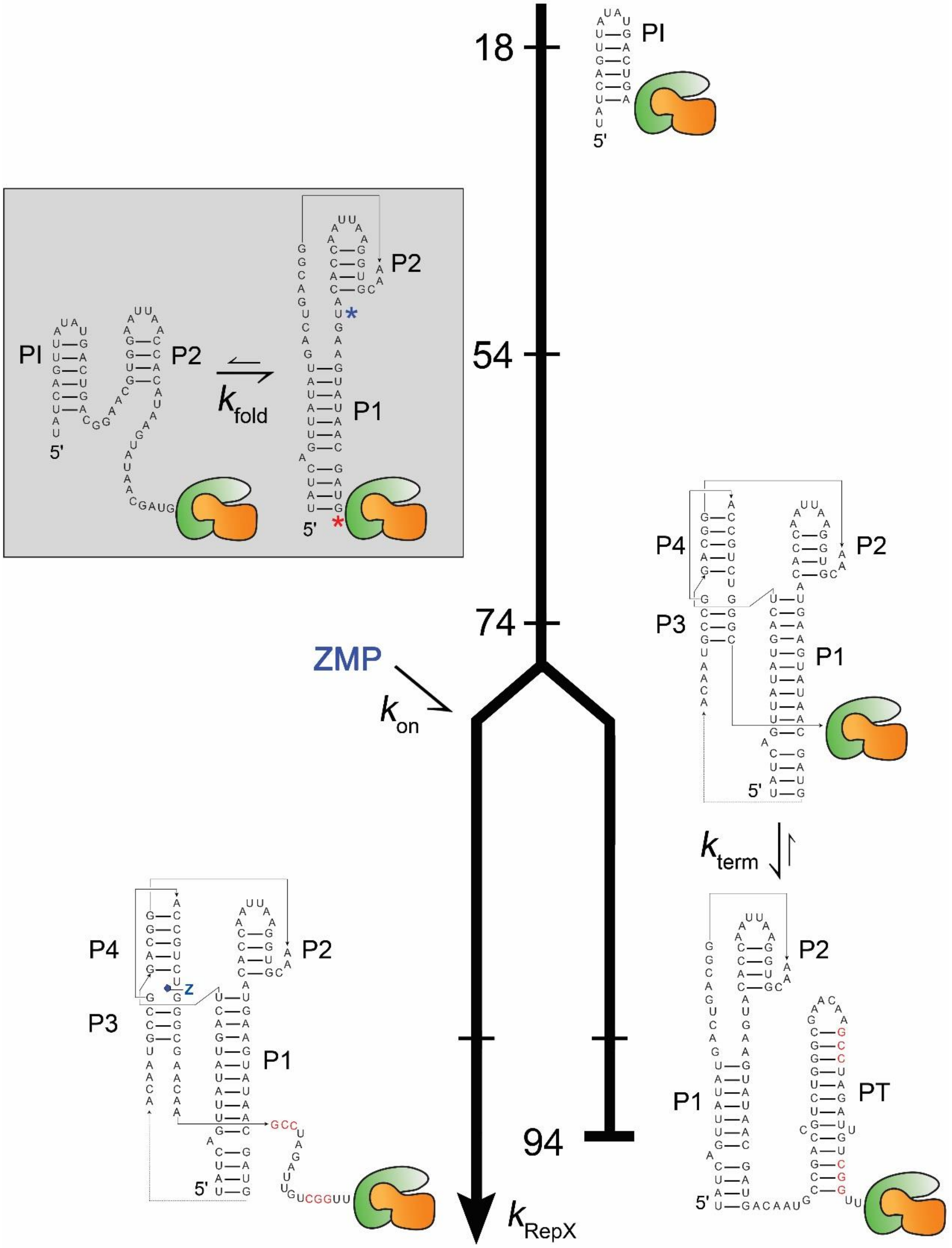

Supplementary Figure 7 Model for ZTP riboswitch folding during helicase unwinding. A sequence axis indicates sequence space for folding of paired elements and sequence position, with proposed secondary structures of RNAs shown below. Rep-X is indicated by an orange and green

cartoon. Folding of intermediate helix PI by position 18 is eventually superseded by folding of helices P1 and P2 at position 54, which are favored by pausing at this position (red asterisk) as opposed to prior to completion of P1 folding (blue asterisk). By position 74, helices P1-P4 can fold and can bind ZMP. If ZMP is present, ZMP binding stabilizes the aptamer fold, which would promote transcription readthrough. If ZMP has not bound, terminator hairpin (PT) folding can begin, completing by position 94, which would promote transcription termination. Nucleotides 81-83 and 92-94 of the terminator hairpin are colored red. The relevant rates are k_{fold}, the folding rate from PI to P1; k_{term}, the folding rate from the aptamer (P1-P4) to the terminator containing P1, P2, PT; and k_{on}, the binding on rate for ZMP. The rate of helicase unwinding ($k_{\mathrm{Rep\text{-}X}}$) dictates the overall rate of movement in sequence space above.

Supplementary Table 1

RNA and DNA oligonucleotide sequences used for making the single-molecule fluorescence constructs.

**Name**	**Sequences**
Ligated *F. ulcerans* ZTP riboswitch (WT)	UAUCAGUUAUAUGACUGACGGAACGUGGAAUUAACCACAUGAAGUAUAACGAUGACAAUGCCGACCGUCUGGGCGAACAAGCCUAGAUUGUCGGUUCAUUAGCGGUAUUCCGGAAUUGCCGUAAUCGCG
Ligated *F. ulcerans* ZTP aptamer (Δterm)	UAUCAGUUAUAUGACUGACGGAACGUGGAAUUAACCACAUGAAGUAUAACGAUGACAAUGCCGACCGUCUGGGCGUUCAUUAGCGGUAUUCCGGAAUUGCCGUAAUCGCG
Ligated *F. ulcerans* ZTP riboswitch (81-83A)	UAUCAGUUAUAUGACUGACGGAACGUGGAAUUAACCACAUGAAGUAUAACGAUGACAAUGCCGACCGUCUGGGCGAACAAAAAUAGAUUGUCGGUUCAUUAGCGGUAUUCCGGAAUUGCCGUAAUCGCG
Ligated *F. ulcerans* ZTP riboswitch (92-94A)	UAUCAGUUAUAUGACUGACGGAACGUGGAAUUAACCACAUGAAGUAUAACGAUGACAAUGCCGACCGUCUGGGCGAACAAGCCUAGAUUGUAAAUUCAUUAGCGGUAUUCCGGAAUUGCCGUAAUCGCG
Ligated *F. ulcerans* ZTP riboswitch (81-83, 92-94A)	UAUCAGUUAUAUGACUGACGGAACGUGGAAUUAACCACAUGAAGUAUAACGAUGACAAUGCCGACCGUCUGGGCGAAC

	AAAAAUAGAUUGUAAAUUCAUUAGCGGUAUUCCGGAAUU<br>GCCGUAAUCGCG
nt 1-33, Cy3 at U32	UAUCAGUUAUAUGACUGACGGAACGUGGAAU-Cy3-UA
nt 1-33, Cy3 at U12	UAUCAGUUA-Cy3-UAUGACUGACGGAACGUGGAAUUA
nt 34-71	ACCACAUGAAGUAUAACGAUGACAAUGCCGACCGUCUG
nt 72-94, Cy3 at U84	GGCGAACAAGCC-Cy3-UAGAUUGUCGGTTCATTAGCGGTA<br>TTCCGGAATTGCCGTAAT CGCG
18-speedmer	GCCUCGCUGCCGUCGCCA-Cy3
biotin-DNA-Cy5	biotin-CGCGATTACGGCAATTCCGGAATACCGCTAATG-Cy5
dT$_{20}$ cDNA to WT	CCGACAATCTAGGCTTGTTCGCCCAGACGGTCGGCATTGT<br>CATCGTTATACTTCATGTGGTTAATTCCACGTTCCGTCAGT<br>CATATAACTGATATTTTTTTTTTTTTTTTTTTT
dT$_{20}$ cDNA to Δterm	CGCCCAGACGGTCGGCATTGTCATCGTTATACTTCATGTGG<br>TTAATTCCACGTTCCGTCAGTCATATAACTGATA<br>TTTTTTTTTTTTTTTTTTTT
dT$_{20}$ cDNA to 81-83A	CCGACAATCTATTTTTGTTCGCCCAGACGGTCGGCATTGTC<br>ATCGTTATACTTCATGTGGTTAATTCCACGTTCCGTCAGTC<br>ATATAACTGATATTTTTTTTTTTTTTTTTTTT
dT$_{30}$ short cDNA to nt 56-94	CCGACAATCTAGGCTTGTTCGCCCAGACGGTCGGCATTG<br>TTTTTTTTTTTTTTTTTTTTTTTTTTTTTT
cDNA-speedmer-dT$_{20}$	CCGACAATCTAGGCTTGTTCGCCCAGACGGTCGGCAT<br>TGTCATCGTTATACTTCATGTGGTTAATTCCACGTTCC

	GTCAGTCATATAACTGATATTTGGCGACGGCAGCGAG GCTTTTTTTTTTTTTTTTTTTT
F. ulcerans ZTP aptamer plasmid[1]	TAATACGACTCACTATAGGGAGATATAACTGATACTGAT GAGTCCGTGAGGACGAAACGGTACCCGGTACCGTCTAT CAGTTATATGACTGACGGAACGTGGAATTAACCACATGA AGTATAACGATGACAATGCCGACCGTCTGGGCG
F. ulcerans ZTP transcription termination plasmid[1]	TTGACTATTTTACCTCTGGCGGTGATAATGGTTGCAATG TAGTAAGGAGGTTGTATGGAAGATTATCAGTTATATGAC TGACGGAACGTGGAATTAACCACATGAAGTATAACGAT GACAATGCCGACCGTCTGGGCGAACAAGCCTAGATTGTC GGTTTTTTTTATACATTTTTTTAGGAGGAGATAATAAGGG AATATCAAATATAATTGTTGA
F. ulcerans ZTP transcription termination plasmid G81A[1]	TTGACTATTTTACCTCTGGCGGTGATAATGGTTGCAATG TAGTAAGGAGGTTGTATGGAAGATTATCAGTTATATGAC TGACGGAACGTGGAATTAACCACATGAAGTATAACGAT GACAATGCCGACCGTCTGGGCGAACAAACCTAGATTGTC GGTTTTTTTTATACATTTTTTTAGGAGGAGATAATAAGGG AATATCAAATATAATTGTTGA
F. ulcerans ZTP transcription termination plasmid 81-83A[1]	TTGACTATTTTACCTCTGGCGGTGATAATGGTTGCAATG TAGTAAGGAGGTTGTATGGAAGATTATCAGTTATATGAC TGACGGAACGTGGAATTAACCACATGAAGTATAACGAT GACAATGCCGACCGTCTGGGCGAACAAAAATAGATTGTC

	GGTTTTTTTTATACATTTTTTTAGGAGGAGATAATAAGGG AATATCAAATATAATTGTTGA
F. ulcerans ZTP transcription termination plasmid 81-86A,87C[1]	TTGACTATTTTACCTCTGGCGGTGATAATGGTTGCAATG TAGTAAGGAGGTTGTATGGAAGATTATCAGTTATATGAC TGACGGAACGTGGAATTAACCACATGAAGTATAACGAT GACAATGCCGACCGTCTGGGCGAACAAAAAAAAACTGTC GGTTTTTTTTATACATTTTTTTAGGAGGAGATAATAAGGG AATATCAAATATAATTGTTGA
Primers to delete nt 1-33 from the plasmid	ACGGTACCCGGTACCGTCACCACATGAAGTATAACG, CGTTATACTTCATGTGGTGACGGTACCGGGTACCGT
Primers to change hammerhead in Δ1-33 plasmid	CGACTCACTATAGGGAGACTTCATGTGGTCTGATGAGT CCGTGAGGAC, GTCCTCACGGACTCATCAGACCACATGAAGTCTCCCTATA GTGAGTCG
PCR T7 promoter forward primer	TAATACGACTCACTATAG
PCR reverse primer to make Δ1-33 with tether RNA for WT	mCmGCGATTACGGCAATTCCGGAATACCGCTAATGAACC GACAATCTAGGCTTGTTCGCCCAGACGGTCGGCAT[2]
PCR reverse primer to make Δ1–33 with tether RNA for Δterm	mGmCGATTACGGCAATTCCGGAATACCGCT AATGAACGCCCAGACGGTCGGCAT[2]

PCR reverse primer to make Δ1-33 with tether RNA for 81-83A	mCmGCGATTACGGCAATTCCGGAATACCGCTA ATGAACCGACAATCTATTTTTGTTCGCCCAGAC GGTCGGCAT[2]
PCR reverse primer to make Δ1-33 with tether RNA for 92-94A	mCmGCGATTACGGCAATTCCGGAATACCGCTA ATGAATTTACAATCTAGGCTTGTTCGCCCAGAC GGTCGGCAT[2]
PCR reverse primer to make Δ1-33 with tether RNA for 81-83, 92-94A	mCmGCGATTACGGCAATTCCGGAATACCGCTA ATGAATTTACAATCTATTTTTGTTCGCCCAGAC GGTCGGCAT[2]

Notes:

[1]For plasmids, only the promoter and transcribed regions are shown.

[2]Nucleotides with 2´-O-methyl modifications are preceded by “m”.
