# Supplementary material for: Real-time monitoring of single ZTP riboswitches reveals a complex and kinetically controlled decision landscape
Source: Nat Commun. 2020 Sep 10;11:4531. doi: 10.1038/s41467-020-18283-1 (PMC7484762; doi:10.1038/s41467-020-18283-1)
Supplement: Supplementary file 3 — Reporting Summary [file 41467_2020_18283_MOESM3_ESM.pdf]

nature research

Corresponding author(s): Taekjip Ha, Adrian R. Ferré-D'Amaré

Last updated by author(s): Jul 20, 2020

Reporting Summary

Nature Research wishes to improve the reproducibility of the work that we publish. This form provides structure for consistency and transparency in reporting. For further information on Nature Research policies, see our Editorial Policies and the Editorial Policy Checklist.

Statistics

For all statistical analyses, confirm that the following items are present in the figure legend, table legend, main text, or Methods section.

n/a	Confirmed	
☐	☒	The exact sample size (n) for each experimental group/condition, given as a discrete number and unit of measurement
☐	☒	A statement on whether measurements were taken from distinct samples or whether the same sample was measured repeatedly
☒	☐	The statistical test(s) used AND whether they are one- or two-sided<br>*Only common tests should be described solely by name; describe more complex techniques in the Methods section.*
☒	☐	A description of all covariates tested
☒	☐	A description of any assumptions or corrections, such as tests of normality and adjustment for multiple comparisons
☐	☒	A full description of the statistical parameters including central tendency (e.g. means) or other basic estimates (e.g. regression coefficient) AND variation (e.g. standard deviation) or associated estimates of uncertainty (e.g. confidence intervals)
☒	☐	For null hypothesis testing, the test statistic (e.g. F, t, r) with confidence intervals, effect sizes, degrees of freedom and P value noted<br>*Give P values as exact values whenever suitable.*
☒	☐	For Bayesian analysis, information on the choice of priors and Markov chain Monte Carlo settings
☒	☐	For hierarchical and complex designs, identification of the appropriate level for tests and full reporting of outcomes
☒	☐	Estimates of effect sizes (e.g. Cohen's d, Pearson's r), indicating how they were calculated

Our web collection on statistics for biologists contains articles on many of the points above.

Software and code

Policy information about availability of computer code

Data collection	Single-molecule data acquisition softwares are custom made (i.e., "smCamera"). They are available at http://ha.med.jhmi.edu/resources/ or upon request.
Data analysis	Single-molecule data analysis softwares are custom made and are available upon request. Peak fitting was done with OriginLab 9.0.

For manuscripts utilizing custom algorithms or software that are central to the research but not yet described in published literature, software must be made available to editors and reviewers. We strongly encourage code deposition in a community repository (e.g. GitHub). See the Nature Research guidelines for submitting code & software for further information.

Data

Policy information about availability of data

All manuscripts must include a data availability statement. This statement should provide the following information, where applicable:

- Accession codes, unique identifiers, or web links for publicly available datasets
- A list of figures that have associated raw data
- A description of any restrictions on data availability

The datasets generated during and/or analyzed during the current study are provided in the source date file and are available from the corresponding authors on reasonable request.

Field-specific reporting

Please select the one below that is the best fit for your research. If you are not sure, read the appropriate sections before making your selection.

- [x] Life sciences
- [ ] Behavioural & social sciences
- [ ] Ecological, evolutionary & environmental sciences

For a reference copy of the document with all sections, see nature.com/documents/nr-reporting-summary-flat.pdf

Life sciences study design

All studies must disclose on these points even when the disclosure is negative.

Sample size	For bulk biochemistry experiments (single-round transcription termination, isothermal titration calorimetry), a sample size of 3 was chosen initially, and experiments were repeated as needed if deviations were large. In cases where no activity was observed (and no deviation could be reported), experiments were repeated three times. For bulk biochemistry experiments, sample size was determined by the number of independent titrations. No statistical analysis was used to predetermine sample sizes, which were chosen based on our past experience with these experiments. For single-molecule experiments, a sample size of 2-4 was chosen for histograms. 15-120 trajectories were obtained for each condition, typically from at least 3 individual experiments. No statistical analysis was used to predetermine sample sizes, which were chosen based on our past experience with these experiments.
Data exclusions	For bulk biochemistry experiments, data was excluded due to technical failure of an experiment (i.e., bubble during ITC titration), which is a pre-established exclusion criterion. For single-molecule experiments measuring two PIFE peaks, traces were excluded if more than two peaks were evident, as noted in the text. Likewise, for traces in which a drop in Cy3 intensity was followed by a PIFE peak, traces were excluded if more than one PIFE was evident, as noted in the text. The exclusion criteria were pre-established in the field to address common technical issues, such as optically overlapping molecules, sub-stoichiometrically labeled samples and etc.
Replication	For for both single-molecule and bulk biochemistry experiments, experiments were performed at least 3 times in independent experiments. Replication was successful for all experiments.
Randomization	Randomization was not performed as it is not customary in this field.
Blinding	Blinding was not performed as it is not customary in this field.

Reporting for specific materials, systems and methods

We require information from authors about some types of materials, experimental systems and methods used in many studies. Here, indicate whether each material, system or method listed is relevant to your study. If you are not sure if a list item applies to your research, read the appropriate section before selecting a response.

Materials & experimental systems

n/a	Involved in the study	
☒	☐	Antibodies
☒	☐	Eukaryotic cell lines
☒	☐	Palaeontology and archaeology
☒	☐	Animals and other organisms
☒	☐	Human research participants
☒	☐	Clinical data
☒	☐	Dual use research of concern

Methods

n/a	Involved in the study	
☒	☐	ChIP-seq
☒	☐	Flow cytometry
☒	☐	MRI-based neuroimaging
